# Neuroprotective mechanisms of valproic acid and alpha-lipoic acid in ALS: a network pharmacology-based investigation

**DOI:** 10.3389/fphar.2025.1681929

**Published:** 2025-10-27

**Authors:** Dongmei Zhang, Ling Han, Wenmo Zhang, Di Wang, Di Huo, Xingli Tan, Xiaoli Su, Ming Wang, Jing Xu, Jiling Cheng, Jing Wang, Honglin Feng

**Affiliations:** ^1^ Department of Neurology, The First Affiliated Hospital of Harbin Medical University, Harbin, Heilongjiang, China; ^2^ School of Pharmaceutical Sciences, Hebei Medical University, Shijiazhuang, Hebei, China

**Keywords:** Amyotrophic lateral sclerosis, valproic Acid, alpha-lipoic acid, network pharmacology, molecular docking, experiment validation

## Abstract

**Introduction:**

Amyotrophic lateral sclerosis (ALS) is a fatal neurodegenerative disorder, and its multi-mechanism pathology makes single-target therapy insufficient. Valproic acid (VPA) and alpha-lipoic acid (ALA) are known neuroprotective agents, but their combined therapeutic potential and mechanisms in ALS remain unclear.

**Methods:**

In this study, network pharmacology method was used to integrate the target data of VPA, ALA and ALS, and key targets and pathways were screened by function enrichment, protein–protein interaction (PPI), network analysis and molecular docking. Furthermore, Mendel randomization (MR) was used to analyze the causal relationship between targets and ALS risk. The synergistic neuroprotective effects of VPA and ALA were then validated in the hSOD1^G93A^ ALS cell and mouse models.

**Results:**

In this study, four core targets-TNF, EGFR, MAPK1 and MAPK8-were identified for the first time. Genetic analysis indicated that higher TNF levels and reduced MAPK8 expression are linked to a greater risk of ALS. Molecular docking demonstrated strong binding affinities of both compounds to these targets. *In vitro* and *in vivo* experiments showed that the combined therapy significantly improved neuronal survival and motor function, inhibited inflammation and apoptosis by activating the PI3K/AKT/FoxO3a pathway, and yielded significantly better therapeutic effects compared to the single drug treatments.

**Discussion:**

VPA and ALA synergistically alleviate ALS by modulating multiple targets and activating the PI3K/AKT/FoxO3a pathway. These findings support their potential as a combinatorial therapeutic strategy for ALS.

## 1 Introduction

ALS is a lethal neurodegenerative disorder marked by the gradual degradation of motor neurons (MN), resulting in muscle weakness, paralysis, and ultimately death (Feldman et al., 2022). While the majority of ALS cases are sporadic (sALS), over 10% are familial (fALS) (Renton et al., 2014), linked to genetic mutations including SOD1, TARDBP, FUS, and C9orf723 (Gurney et al., 1994). Despite considerable advancements in elucidating the pathogenesis of ALS, the available therapeutic options remain limited, with only four U.S. Food and Drug Administration-approved agents for ALS management: riluzole, edaravone, Relyvrio (Arnold et al., 2024), and Tofersen (Blair, 2023). Although these drugs provide some benefits to certain ALS patients, their efficacy is restricted, and they do not address the core pathological mechanisms of ALS effectively. Numerous variables, like oxidative stress, protein aggregation, neuroinflammation, excitotoxicity, and mitochondrial dysfunction, contribute to the pathophysiology of ALS (Mead et al., 2023). Addressing a single pathway is often inadequate for effective treatment, underscoring the necessity of developing multi-target strategies. In this regard, VPA and ALA have garnered attention for their neuroprotective properties and potential therapeutic benefits in ALS.

VPA, a histone deacetylase (HDAC) inhibitor and anticonvulsant with excellent blood-brain barrier permeability, is widely used for treating epilepsy and bipolar disorder (Chateauvieux et al., 2010). Its neuroprotective effects include anti-inflammatory, anti-apoptosis and regulation of neurotrophic factor expression (Huang et al., 2014; Khan et al., 2015; Rouaux et al., 2007; Sugai et al., 2004). Although long-term use may lead to hepatotoxicity, teratogenicity or neurotoxicity due to the production of reactive oxygen species (ROS), VPA still shows remarkable efficacy in various neurodegenerative disease models and is regarded as a promising candidate drug (Chiu et al., 2013; Wang et al., 2024). With regard to ALS, VPA may improve pathology through multiple mechanisms, including the modulation of transcription factors, regulation of splicing factor expression, inhibition of glutamate toxicity, and modulation of autophagy (Wang et al., 2015). Our previous investigations have demonstrated that co-treatment with VPA and lithium can mitigate mitochondrial dysfunction and MN death induced by oxidative stress by upregulating SIRT3 and CARM1 (Wang et al., 2013). Additionally, it inhibits Homer1b/c upregulation induced by the SOD1‐G93A mutation, thereby alleviating ALS-associated neuronal apoptosis (Jiang et al., 2016).

ALA is a natural antioxidant, which is very important for mitochondrial function. Owing to its antioxidative and anti-inflammatory attributes, ALA has exhibited neuroprotective capabilities in various neurodegenerative disorders by eliminating ROS, binding metal ions, and regulating signaling pathways linked to oxidative stress and inflammatory responses (Choi et al., 2015; Packer et al., 1997; Tripathi et al., 2023; Wang et al., 2018a). Our team has systematically validated in both hSOD1^G93A^ cell models and *Drosophila* models that ALA exerts neuroprotective effects through the activation of the ERK-Akt pathway (Wang, 2018).

Given the complementary neuroprotective mechanisms of VPA and ALA, their combination may enhance therapeutic efficacy against ALS. Network pharmacology provides a theoretical framework for the design of combined therapy by integrating systems biology and bioinformatics, focusing on multi-target and multi-channel interaction (Sidhu and Capalash, 2021; Wang et al., 2021). It has been widely used in neurodegenerative diseases such as Alzheimer’s disease, but its application is still limited in the field of ALS (Zhu et al., 2023). At present, ALS-related research mostly focuses on Traditional Chinese Medicine (TCM) compounds (such as Rehmannia glutinosa, Strychnos nux-vomica, Jianpi Bushen formula, *etc.*) (Li et al., 2023; Tang et al., 2023; Lin et al., 2022). Network pharmacology, which combines molecular docking (Santos et al., 2019) and MR (Khasawneh et al., 2022; Xiao et al., 2024), can promote the identification of new molecular targets and causality in ALS, thereby advancing the development of precise treatment strategies.

Currently, no studies have utilized network pharmacology to investigate the underlying processes of the combined VPA and ALA therapy in ALS. ALA has demonstrated neuroprotective effects by mitigating oxidative stress and preventing VPA-induced brain damage in preclinical models (Turkyilmaz et al., 2020). Considering their distinct yet complementary properties, it is essential to investigate their potential synergistic effects in ALS. This study seeks to examine the synergistic and complementary effects of VPA and ALA in ALS using network pharmacology, bioinformatics, MR analysis, molecular docking, and experimental verification. By identifying key targets and pathways, our research will provide a scientific basis for ALS multi-target drug design, promote the integration of computational prediction and experimental verification, and accelerate the clinical application of combination therapy guided by network pharmacology.

## 2 Materials and methods

### 2.1 Identification of the targets of VPA and ALA

**FIGURE 1:**
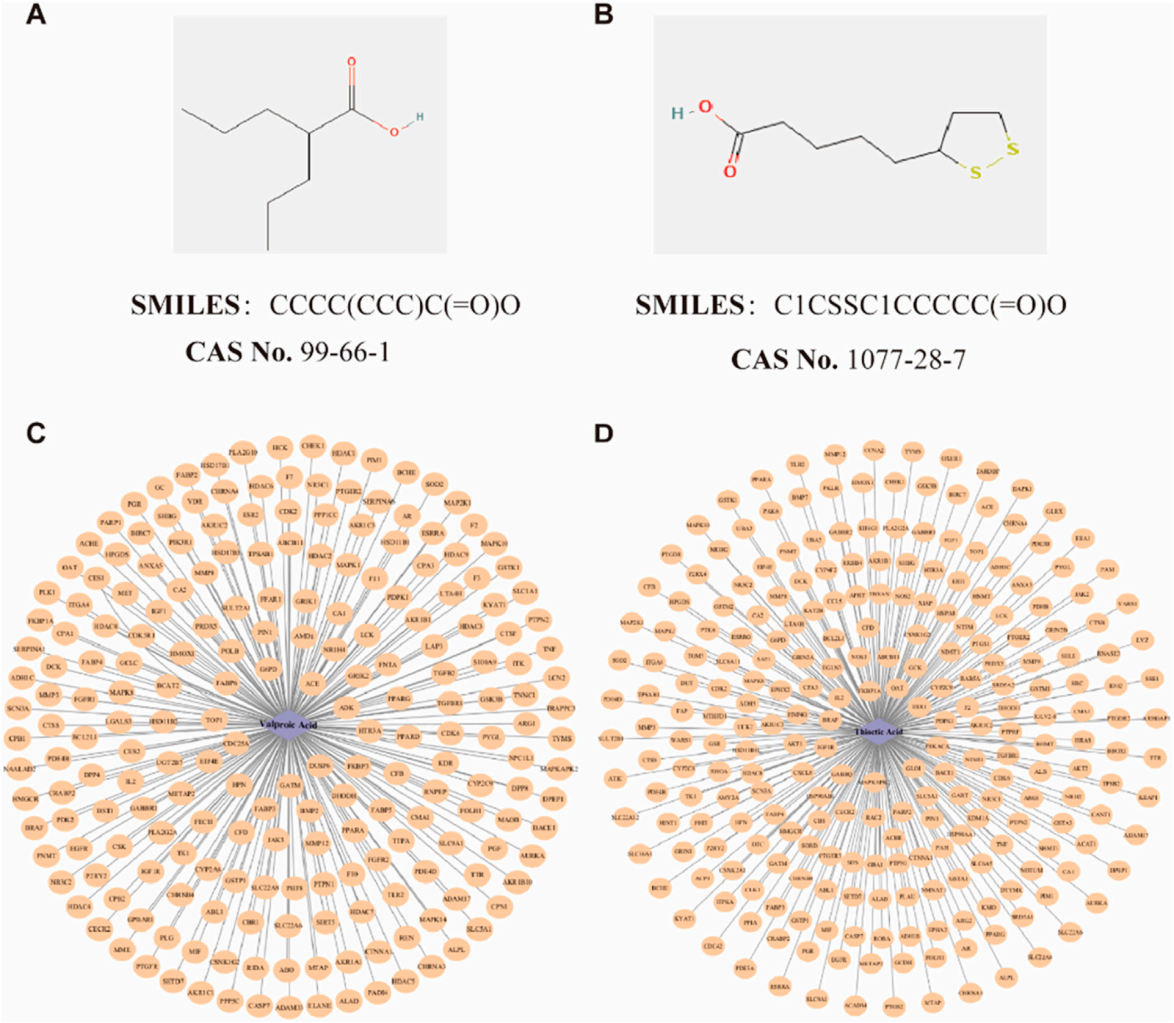
Potential targets identification of VPA and ALA. Note: The detailed information of VPA **(A)** and ALA **(B)**, as well as the drug-target interaction network of VPA **(C)** and ALA **(D)**.

A thorough search was conducted in the PubChem database (Kim et al., 2022) (https://pubchem.ncbi.nlm.nih.gov/) utilizing the keywords “Valproic acid” and “Alpha-Lipoic acid” (or its synonym “Thioctic acid”) to retrieve the Simplified Molecular Input Line Entry System (SMILES) representations, Chemical Abstracts Service (CAS) numbers, ChEMBL IDs, and Mol2 format files of VPA (CAS: 99-66-1) and ALA (CAS: 1077-28-7) (Figures 1A,B). The SMILES strings were subsequently input into Binding DB (Gilson et al., 2016) (https://www.bindingdb.org/), Similarity Ensemble Approach (SEA) (Keiser et al., 2007) (https://sea.bkslab.org/), SwissTargetPrediction (Daina et al., 2019) (http://swisstargetprediction.ch/), Chembl (Zdrazil et al., 2023) (https://www.ebi.ac.uk/chembl/), and PharmMapper (Wang et al., 2017) (http://lilab-ecust.cn/pharmmapper/) to predict potential drug targets.

To enhance the relevance of targets, the following selection criteria were applied: In SwissTargetPrediction, targets with a probability >0 were considered. Targets with a confidence score of ≥90% were included in ChEMBL. In PharmMapper, targets were included if the z-score >0. The species in every database was limited to “*Homo sapiens*.” After collecting potential targets, they were cross-referenced with UniProt (Coudert et al., 2022) to standardize protein annotations. Duplicate entries were removed to generate the final list of predicted targets for VPA and ALA.

### 2.2 Identification of the targets of ALS

Gene targets associated with the disease were retrieved by searching the keyword “Amyotrophic Lateral Sclerosis” in the following databases: GeneCards (Stelzer et al., 2016) (https://www.genecards.org/), OMIM (Amberger et al., 2019) (https://omim.org/), DisGeNET (Piñero et al., 2020) (https://www.disgenet.org/search) and DrugBank (Wishart et al., 2018) (https://go.drugbank.com/). A relevance score >3 was applied as a screening criterion in the GeneCards database. The retrieved gene targets were then standardized by converting them into official gene symbols using the UniProt database.

Two microarray datasets regarding ALS, GSE833 and GSE4595, were acquired from the Gene Expression Omnibus (GEO) database (Barrett et al., 2013) (www.ncbi.nlm.nih.gov/geo/). GSE833 comprises spinal cord samples from four control subjects and seven ALS patients (Dangond et al., 2004), while GSE4595 includes cortical samples from 9 controls and 11 ALS patients (Lederer et al., 2007). Differentially expressed genes (DEGs) were found through the analysis of ALS and control samples utilizing the edgeR and limma tools in R (version 4.4.0). Genes with |logFC|>1 and P < 0.05 were considered significant in statistics.

The ALS-related genes from GeneCards, OMIM, DisGeNET, and DrugBank were then integrated with the GEO microarray-derived DEGs. After removing duplicate entries, a final set of potential ALS disease targets was obtained.

### 2.3 Identification of overlapping targets among VPA, ALA, and ALS

Through Venn diagram analysis, we identified the overlapping targets of VPA, valproic acid ALA and ALS. These overlapping targets were then further examined to explore the potential synergistic and complementary effects of VPA and ALA in ALS therapy. A Venn diagram was created for visualization through Bioinformatics (http://www.bioinformatics.com.cn/).

### 2.4 GO and KEGG enrichment analysis

In this study, the clusterProfiler package in R language was used to analyze the function enrichment of potential target genes, including Gene Ontology (GO) (The Gene Ontology Resource, 2019) and Kyoto Encyclopedia of Genes and Genomes (KEGG) (Kanehisa et al., 2016) pathway analysis. GO analysis covers three dimensions: Biological Process (BP), Cellular Component (CC) and Molecular Function (MF). The annotation database is org. Hs.e.g.,.db (v3.18.0), which ensures the accurate mapping between human gene symbols and Entrez ID. Visualization uses enrichplot to generate bubble chart, bar chart and path network chart, ggplot2 is used to customize color matching, labels and layout to highlight key paths.

Through the analysis of GO terms, the biological functions of potential targets are deeply analyzed. KEGG pathway analysis is based on KEGG. db built in clusterProfiler and online API, focusing on pathways related to neurodegenerative diseases, apoptosis, inflammation and oxidative stress. In order to reduce the risk of false positives caused by multiple hypothesis testing and enhance the reliability of the results, Benjamini–Hochberg (BH) method was used to correct the false detection rate (FDR), and the significance threshold was set to p_adjust <0.05.

### 2.5 Construction of the PPI network

To investigate the synergistic and complementary effects of VPA and ALA in ALS therapy, we imported the identified targets into the STRING database (Szklarczyk et al., 2022), selecting “*H. sapiens*” as the organism and using a “high confidence” criterion. The PPI network was later displayed, further analyzed, and key targets were discovered utilizing Cytoscape (Version 3.10.2) (Reimand et al., 2019).

### 2.6 Determination of core targets for the synergistic effects of VPA and ALA

The PPI network, comprising 68 synergistic targets of VPA, ALA, and ALS, was loaded into Cytoscape for further examination. Topological analysis was conducted with the CytoHubba (Chin et al., 2014) plugin, which assessed Betweenness, Closeness, and Degree centrality to identify crucial core genes. Furthermore, gene clustering analysis was performed using the MCODE (Bader and Hogue, 2003) plugin with the applied parameters: Degree Cutoff = 2, Node Score Cutoff = 0.4, K-Core = 2, Max Depth from Seed = 100.

To evaluate the functional importance of the specified targets, we constructed a target-pathway interaction network using Cytoscape, incorporating KEGG-enriched pathways and their corresponding target genes, which were subsequently ranked by degree. The core targets for the synergistic effects of VPA and ALA in ALS treatment were determined by identifying intersecting targets across the three analytical methods. A Venn diagram was created for enhanced visualization.

### 2.7 Molecular docking of VPA and ALA with core targets

The candidate chemicals were sourced from the PubChem database. Meanwhile, the PDB (https://www.pdb.org/) provided the targets’ 3D structures. Protein preparation utilized AutoDock Tools 1.5.6, incorporating the addition of hydrogen atoms to the proteins and the assignment of rotatable bonds to the small molecules.

Molecular docking was implemented with AutoDock Vina 1.2.0 (Trott and Olson, 2010), where docking parameters were configured using the Grid module, employing a semi-flexible docking strategy. During the docking phase, the receptor-ligand binding was optimized using the Lamarckian genetic algorithm. The docking results were assessed based on binding free energy, where lower binding energy indicates stronger receptor-ligand affinity and greater stability of the receptor-ligand complex. The docking results were visualized with PyMOL 2.3.0 and Discovery Studio 2019 to facilitate further structural analysis and interpretation.

### 2.8 MR analysis of core targets and ALS risk

This study employed MR analysis to assess the potential causal relationship between ALS risk and primary targets identified through network pharmacology. Data from a genome-wide association study (GWAS) on ALS, including 12,577 cases and 23,475 controls, were sourced from ebi-a-GCST004692 (https://www.ebi.ac.uk/gwas/) (van Rheenen et al., 2016). Single Nucleotide Polymorphisms (SNPs) associated with core target genes were extracted from the eQTLGen database, a large-scale eQTL study incorporating over 30,000 blood samples (Võsa et al., 2021). SNPs were picked using a criteria of P < 5 × 10^−8^, and linkage disequilibrium was controlled at *r* ^2^ < 0.1 within a 500 kb window. Significant SNPs that were present in both eQTLGen and GWAS data, excluding palindromic variants, were retained as candidate instrumental variables (IVs). To minimize weak instrument bias, SNPs with an F-statistic (F = β^2^/SE^2^) <10 were excluded.

Two-sample Mendelian Randomization analysis was performed using R software with the TwoSampleMR package (version 0.6.8). The principal estimate approach utilized was inverse-variance weighted (IVW). The robustness of the results was validated through MR-Egger, weighted median, weighted mode, and Wald ratio (Xie et al., 2023), with a cutoff for significance set at P < 0.05. Cochran’s Q test (Hemani et al., 2018), MR-Egger intercept, and MR-PRESSO (Bowden et al., 2016) were utilized to assess heterogeneity and pleiotropy, confirming the validity of IVs. Finally, forest plots were generated to visually represent the causal estimates.

### 2.9 Cell culture

The NSC34 cell line, a hybrid of murine neuroblastoma cells and embryonic spinal motor neurons, was kindly supplied by Cedarlane Laboratories (Vancouver, Canada). Renowned for its motor neuron-like properties, this cell line has been extensively employed in investigating motor neuron functions associated with ALS. The ALS cell model was established by transfecting NSC34 cells with puromycin-resistant lentiviral vectors carrying hSOD1-G93A, hSOD1-wild-type (WT), or an empty GFP control vector (EV), followed by selection with 2 μg/mL puromycin (Beyotime Institute of Biotechnology, Beijing, PR China) to generate three stable cell lines. The cells were cultured in Dulbecco’s Modified Eagle Medium (DMEM) (Thermo Fisher Scientific, United States), supplemented with high glucose, 10% fetal bovine serum (FBS), and 1% penicillin-streptomycin. Incubation occurred at 37 °C in a humidified environment containing 5% CO_2_. When cell confluence reached approximately 80%, various reagents were applied to the cells for subsequent experimental procedures.

### 2.10 Cell viability assay

Cell viability was assessed using the Cell Counting Kit-8 (Nanjing Xinsaimei, China). The cells were seeded into 96-well plates and cultured overnight in DMEM with 10% FBS. The hSOD1^G93A^ NSC34 cells were then treated with VPA, ALA, or their combination for 24 h. The VPA group included concentrations of 0.5 mM, 0.75 mM, 1 mM, 1.5 mM, and 2 mM, while the ALA group consisted of 10 μM, 50 μM, 100 μM, 125 μM, and 250 μM. The VPA + ALA combination group contained six pairs of concentrations: 1 mM VPA with 50 μM, 100 μM, or 125 μM ALA; and 1.5 mM VPA with 50 μM, 100 μM, or 125 μM ALA. After treatment, 10 μL of CCK-8 solution was added, and cells were incubated at 37 °C for 1 h. Absorbance at 450 nm was measured using a microplate reader (BioTek Instruments, Winooski, VT, United States).

### 2.11 Mouse model and treatment

Transgenic hSOD1^G93A^ mice (B6SJL-Tg-SOD1-G93A-1Gur/J) were sourced from Jackson Laboratory (Bar Harbor, Maine, United States). Animal procedures were authorized by Harbin Medical University’s Experimental Animal Ethics Committee (China) and carried out in accordance with international animal care recommendations. Transgenic hSOD1^G93A^ male mice were obtained by breeding with C57BL/6J mice (Vital River Laboratory Animal Technology Co., Beijing, China), and genotyping was performed by Polymerase Chain Reaction on tail Deoxyribonucleic Acid, DNA. Littermates lacking the hSOD1^G93A^ transgene were used as WT controls. Age-matched male mice were randomly divided into five groups (n = 6 per group, i. p.): WT (saline), hSOD1^G93A^ control (saline), hSOD1^G93A^ VPA (300 mg/kg/day), hSOD1^G93A^ ALA (50 mg/kg/day), and hSOD1^G93A^ VPA + ALA (300 mg/kg/day VPA +50 mg/kg/day ALA). All treatments were administered daily at a fixed time, starting from postnatal day 60 until the study endpoint. The dosage of VPA is based on Feng et al. (2008), while the dosage of ALA is based on Zhang et al. (2023).

Daily measurements of each mouse’s body weight were taken at 9:00 a.m. before intraperitoneal drug administration and were continuously monitored until all mice had succumbed. Beginning at 90 days of age, all transgenic mice were assessed three times per week at a fixed time using the ALS Therapy Development Institute (ALSTDI) neurological scoring system (0–4 points) (Li et al., 2015). The onset of disease was determined as the initial occurrence of a neurological score of 1 in a mouse, whereas end-stage disease or death was characterized by achieving a neurological score of 4. For each mouse, the date of disease onset, date of death, and cause of death were documented.

To assess motor performance, the rotarod test was conducted at a constant speed (16 rpm) to evaluate motor coordination and balance. Starting at approximately 70 days of age, mice underwent a 5-min daily training session for 1 week. Following the training period, testing was conducted every 4 days. The duration for which each mouse remained on the rotating rod before losing balance was documented, with a maximum limit of 180 s. To ensure accuracy, each mouse participated in three trials, and the average duration was computed for further analysis.

Gait analysis was initiated at 80 days of age and performed at 10-day intervals. Mice were placed in an acrylic corridor measuring 50 cm in length and 5 cm in width, lined with white paper. To visualize footprints, the hind paws were coated with non-toxic ink. Each mouse was positioned at one end of the corridor and gently encouraged to walk straight toward the opposite end. From each trial, three consecutive, steady steps (excluding the initial steps) were selected, and the distances between successive hind paw prints were measured and averaged to calculate the step length (Zhan et al., 2025).

### 2.12 Western blotting (WB)

Protein extraction from 130-day mouse tissues was performed using Western and IP lysis buffers (Beyotime, China), supplemented with PMSF (Beyotime, China) and a phosphatase inhibitor cocktail (Roche, Switzerland). The supernatant was collected by centrifuging the tissue lysates at 13,500 rpm for 15 min at 4 °C after incubation on ice for 30 min. Protein concentrations were measured using a BCA protein assay kit (Beyotime, China). The collected supernatant was mixed with loading buffer and heated at 100 °C for 5 min to denature the proteins. Protein samples were separated by Sodium Dodecyl Sulfate-PolyAcrylamide Gel Electrophoresis and subsequently transferred onto Polyvinylidene Difluoride membranes (Millipore, Burlington, VT, United States). Membranes were blocked with 5% non-fat milk and incubated overnight at 4 °C with primary antibodies from various suppliers. Proteintech antibodies included anti-Bcl-2 (66799-1-Ig, 1:2,000), anti-Bax (50599-2-Ig, 1:1,000), anti-Caspase3/p17/p19 (19677-1-AP, 1:2,000), anti-TNF-alpha (17590-1-AP, 1:1000), anti-IL-beta (26048-1-AP, 1:2,000), and anti-hSOD1 (10269-1-AP, 1:10,000). Affinity Biosciences antibodies consisted of anti-PI3K (AF6241, 1:1,000), anti-P-FOXO3A (Ser253) (AF3020, 1:1,000), and anti-FOXO3A (6020, 1:1000). Antibodies from Cell Signaling Technology (CST) included anti-P-AKT (Ser473) (4060T, 1:1,000) and anti-AKT (4691T, 1:1,000). As a loading control, anti-β-actin (ab8227, 1:5,000) was obtained from Abcam. After primary antibody incubation, the membranes were washed and then incubated with secondary antibodies (goat anti-mouse or anti-rabbit IgG conjugated to Alexa Fluor 800; Li-COR, United States, 1:10,000) for 1 h at room temperature. Protein signals were visualized using the Odyssey infrared imaging system (Li-COR, United States), and the band intensities were quantified using ImageJ software (National Institutes of Health, Bethesda, MD, United States), with β-actin serving as the internal control for normalization.

### 2.13 Statistical analysis

All experiments were done in triplicate. The statistical analysis was performed using GraphPad Prism 8.0 (GraphPad Software, San Diego, CA, United States). Data are presented as mean ± standard deviation (SD). The log-rank test (Kaplan-Meier method) was employed to assess ALS onset and survival curves. Tukey’s *post hoc* test was implemented to ascertain significance in comparisons between distinct groups, following the implementation of one-way or two-way analysis of variance (ANOVA). A P-value of less than 0.05 was deemed statistically significant.

## 3 Results

### 3.1 Potential targets identification of VPA and ALA

By predicting the drug targets of SMILES molecular formula of VPA and ALA, we integrated the data and eliminated the duplicates, and finally determined 215 targets of VPA and 249 targets of ALA. The selected target data were imported into Cytoscape software, and a drug-target interaction network was constructed (Figures 1C,D).

### 3.2 Potential targets identification of ALS

**FIGURE 2:**
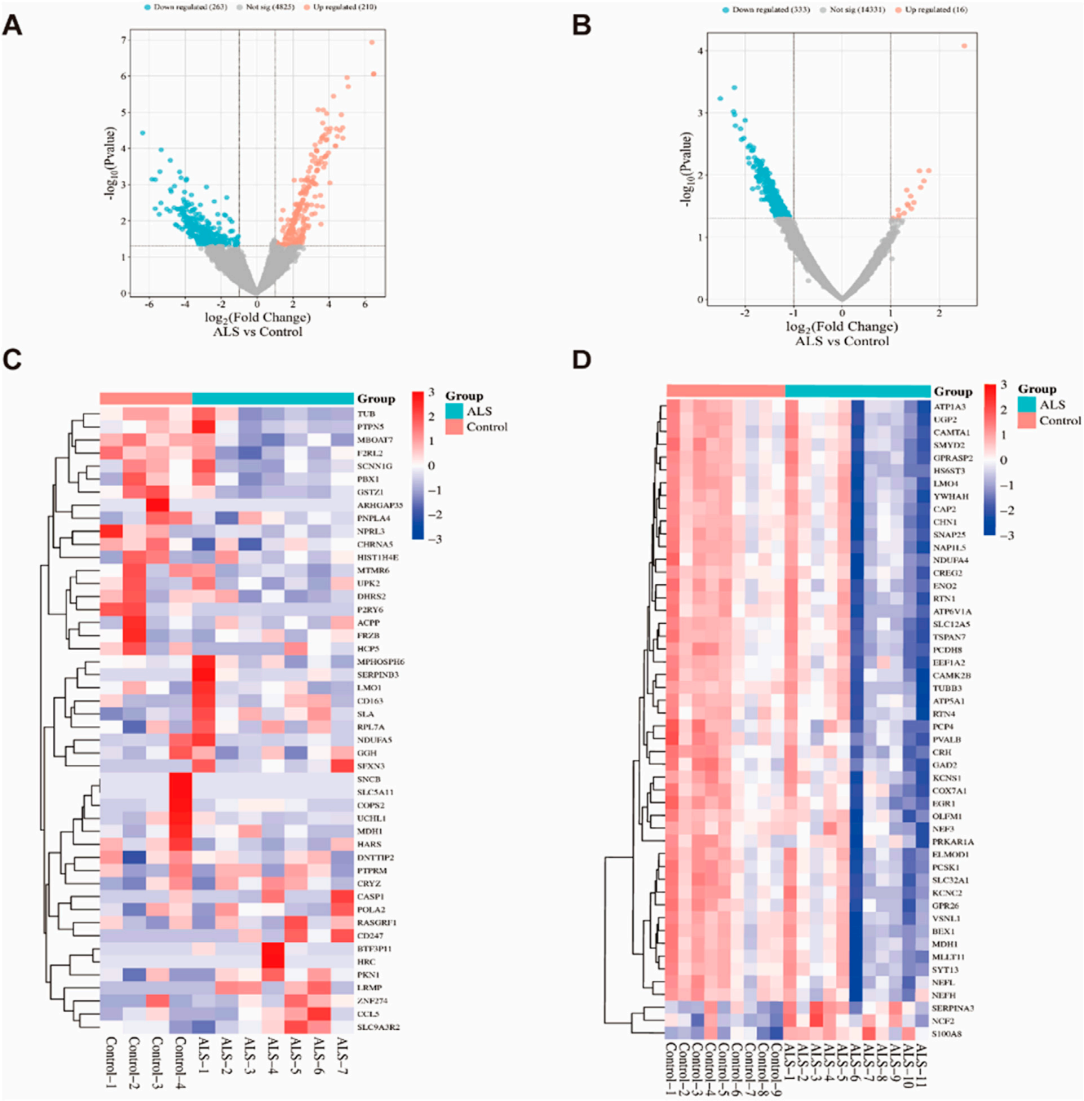
DEGs analysis of the GSE833 and GSE4595 datasets. Note: Volcano plots of DEGs from the GSE833 **(A)** and GSE4595 **(B)** datasets; Heatmaps of the top 50 significantly expressed DEGs from GSE833 **(C)** and GSE4595 **(D)** datasets.

Using the edgeR and limma packages in R, we identified DEGs between the control group and ALS patients. In the GSE833 and GSE4595 datasets, we identified a total of 5,298 and 14,680 DEGs, respectively. In the GSE833 dataset, 210 genes exhibited upregulation, whereas 263 genes showed downregulation (Figure 2A). The GSE4595 dataset showed 16 genes elevated and 333 genes downregulated (Figure 2B). The heatmaps illustrating the top 50 DEGs from the GSE833 and GSE4595 datasets are shown in Figures 2C,D, respectively.

**FIGURE 3:**
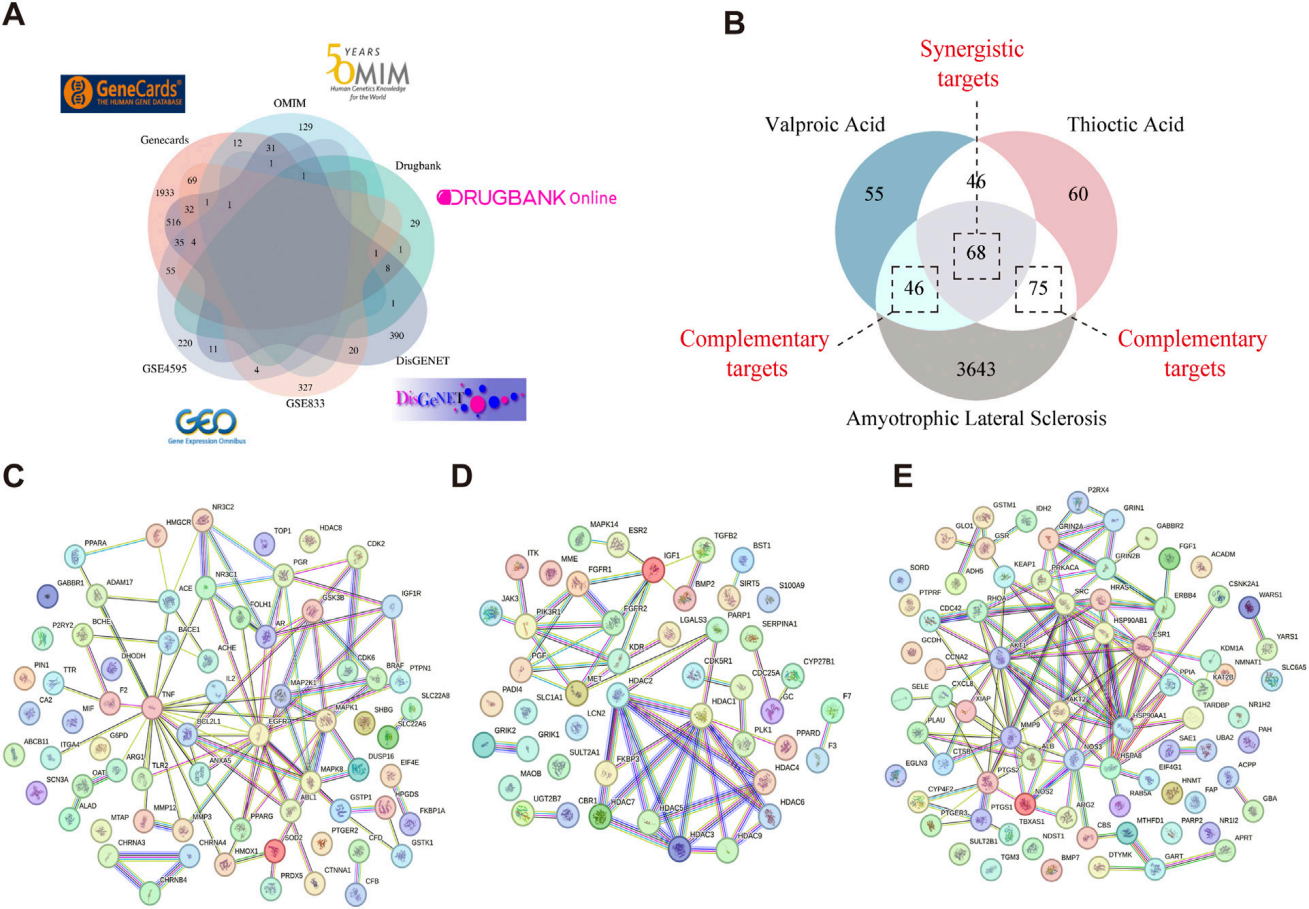
Identification of target genes involved in VPA-ALA-ALS interactions. Note: **(A)** ALS-related targets were retrieved from four distinct databases and GEO microarray analysis. **(B)** Venn diagram illustrating the overlap among VPA, ALA, and ALS-related targets. **(C)** PPI network of 68 synergistic targets of VPA and ALA in ALS. **(D)** PPI network of 46 complementary targets associated with VPA in ALS. **(E)** PPI network of 75 complementary targets associated with ALA in ALS.

ALS-related targets were identified by retrieving 2,701 genes with a relevance score >3 from the GeneCards database. Additionally, ALS-associated targets were obtained from OMIM (176 targets), DisGeNET (1,053 targets), and DrugBank (45 targets). The disease-associated targets from GeneCards, OMIM, DisGeNET, and DrugBank were integrated with the DEGs identified through GEO microarray analysis, and duplicates were eliminated. This resulted in a final set of 3,832 potential ALS-related targets (Figure 3A).

### 3.3 Synergistic and complementary targets of VPA and ALA in ALS: PPI network analysis

Through the Venn diagram, we identified the overlapping targets of VPA, ALA, and ALS, which are considered potential targets for the combination therapy of VPA and ALA in ALS (Figure 3B). We identified 68 gene targets shared by VPA, ALA, and ALS, along with 46 potential complementary targets for VPA and 75 potential complementary targets for ALA in ALS treatment. Investigating these target genes will be necessary to elucidate the neuroprotective mechanisms of VPA and ALA in ALS. To explore the interactions among these targets, both synergistic and complementary targets of VPA and ALA for ALS treatment were submitted to the STRING database for constructing a PPI network (Figures 3C–E).

### 3.4 GO and KEGG analysis of potential targets related to VPA, ALA, and ALS

**FIGURE 4:**
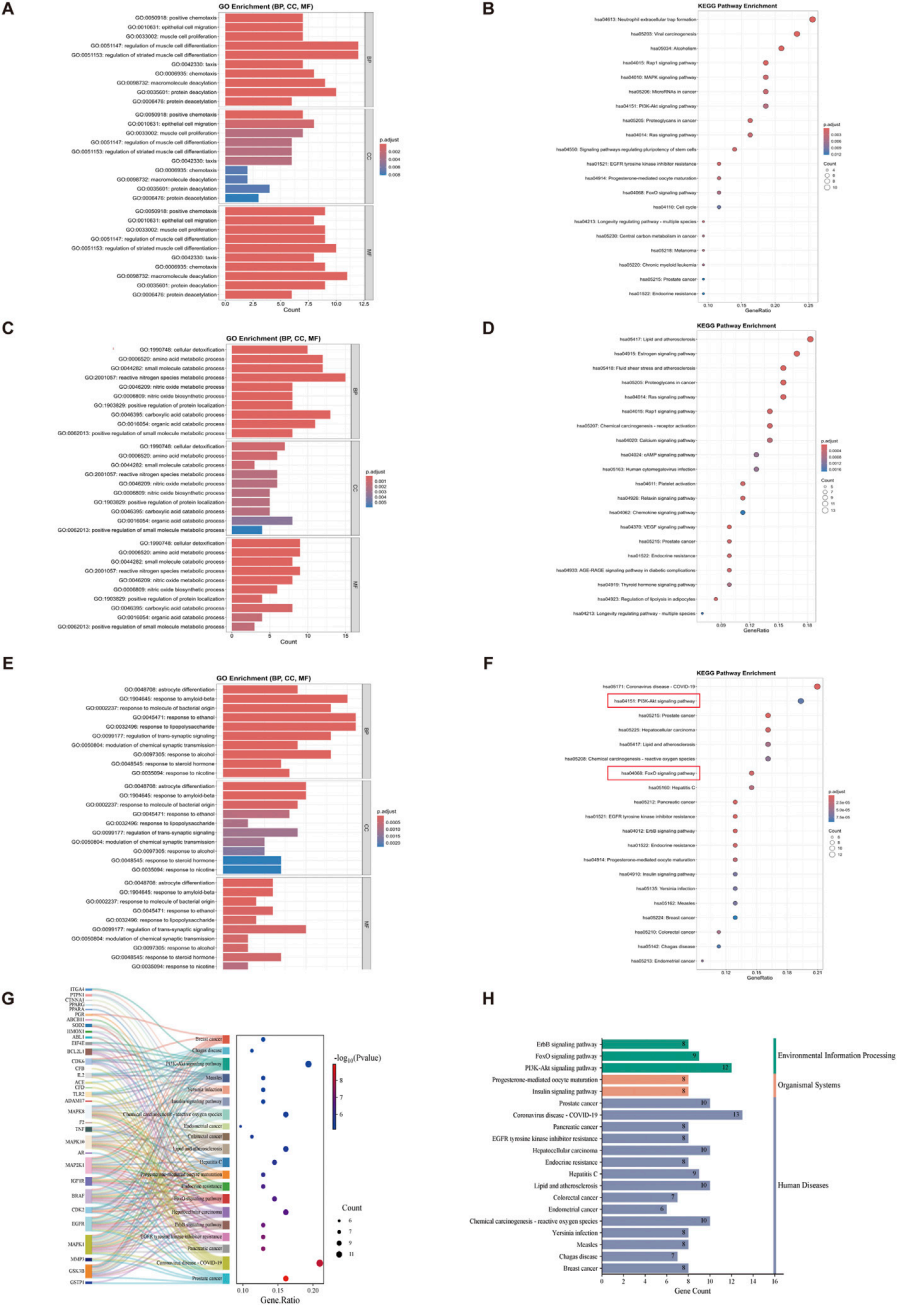
GO and KEGG analysis of synergistic and complementary targets of VPA and ALA combination therapy for ALS. Note: **(A)** Bar chart and **(B)** bubble chart displaying the top 10 most significant GO terms and the top 20 KEGG pathways of potential complementary targets of VPA in ALS; **(C,D)** potential complementary targets of ALA; **(E,F)** potential synergistic targets of VPA and ALA. **(G)** Sankey diagram representing the top 20 KEGG pathways associated with synergistic targets of VPA and ALA. The size of each bubble represents the quantity of genes enriched in the corresponding pathways, and the color intensity indicates the related p-value. **(H)** KEGG first-level classification of these pathways. The color gradients in the bubble and bar charts correspond to p. adjust values, with darker shades indicating higher significance and larger bubbles representing a greater number of enriched genes.

GO enrichment analysis was performed on 46 potential targets tied to the complementary effects of VPA therapy for ALS, leading to the identification of 733 GO terms. Among these, 651 terms were related to BP, 21 terms to CC, and 61 terms to MF. Figure 4A displays bar charts illustrating the ten most enriched GO concepts for each group. The GO enrichment analysis suggests that VPA may exert neuroprotective and pathological repair effects in ALS through mechanisms such as protein modification (e.g., deacetylation), cell migration (e.g., epithelial cell migration), and cell differentiation (e.g., myocyte differentiation and proliferation). The KEGG enrichment analysis of the 46 complementary targets associated with VPA treatment in ALS identified 38 pathways with significant enrichment. Figure 4B presents the top 20 pathways, which include neutrophil extracellular trap formation, viral carcinogenesis, alcoholism, the Rap1 signaling pathway, EGFR tyrosine kinase inhibitor resistance, the MAPK signaling pathway, the PI3K-Akt signaling pathway, and the FoxO signaling pathway.

Similarly, for the 75 potential targets related to the complementary effects of ALA treatment, GO enrichment analysis identified 935 GO terms, including 824 BP-related terms, 36 CC-related terms, and 75 MF-related terms. Figure 4C illustrates the ten most significant terms for each group. The GO analysis indicates that ALA may exert protective effects on ALS by regulating metabolism (e.g., small molecule metabolism, organic acid catabolism), enhancing antioxidant capacity (e.g., nitric oxide metabolism, cellular detoxification), and improving neurotransmission (e.g., glutamatergic synapse, G-protein signaling). The KEGG analysis of the 75 complementary targets of ALA therapy revealed 103 enriched pathways. The top 20 pathways, which include the estrogen signaling system, VEGF signaling pathway, Ras signaling pathway, cAMP signaling pathway, and chemokine signaling pathway, are depicted in Figure 4D.

For the 68 potential targets involved in the synergistic effects of VPA and ALA combination therapy, 1038 GO terms were identified, with 942 BP-related terms, 21 CC-related terms, and 75 MF-related terms. Figure 4E illustrates the ten most prominent terms for each category. The GO analysis results indicate that the potential targets of VPA and ALA combination therapy for ALS primarily involve stress response (e.g., response to nicotine, corticosteroids, lipopolysaccharides, alcohol, *etc.*), regulation of neurotransmission (e.g., chemical synaptic transmission, trans-synaptic signaling regulation), and cell differentiation (e.g., astrocyte differentiation). For the 68 synergistic effect targets of VPA and ALA combination therapy, 117 KEGG pathways were identified. The top 20 pathways (Figure 4F) include prostate cancer, COVID-19, pancreatic cancer, EGFR tyrosine kinase inhibitor resistance, ErbB signaling pathway, PI3K-Akt signaling pathway, and FoxO signaling pathway. A Sankey bubble plot was constructed to illustrate the association between these KEGG pathways and their enriched genes (Figure 4G).

**FIGURE 5:**
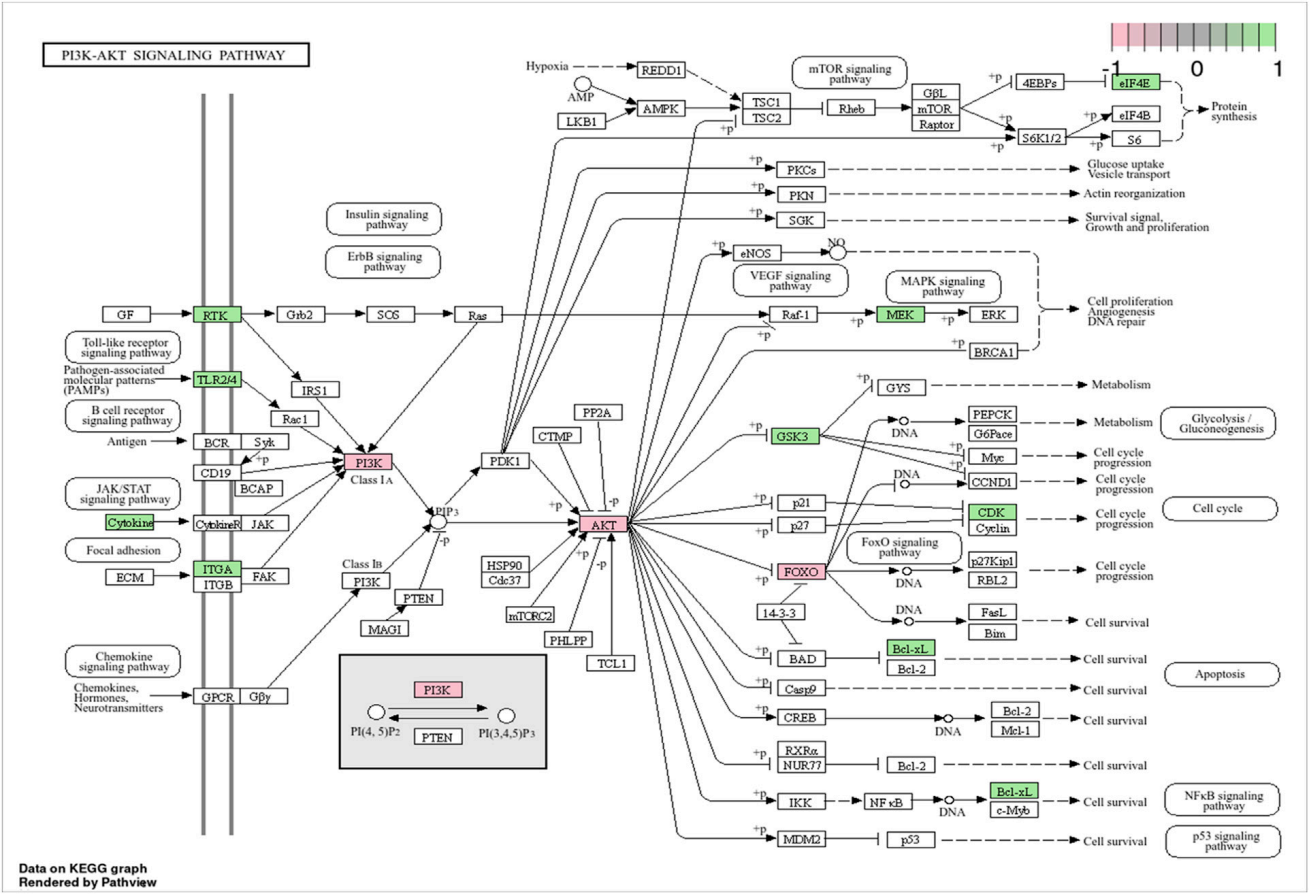
Visualization of the PI3K-AKT signaling pathway. Note: Genes specifically involved in the PI3K/AKT/FoxO axis are highlighted in pink, while those enriched in the PI3K-AKT signaling pathway are marked in green.

KEGG classification was carried out to systematically categorize the biological relevance of the top 20 pathways enriched in synergistic targets. These pathways were classified into three primary categories, as shown in Figure 4H, with the Environmental Information Processing category including the PI3K-Akt, FoxO, and ErbB signaling pathways. The PI3K-AKT, FoxO, and ErbB signaling pathways are interconnected in the PI3K-AKT signaling pathway diagram (Figure 5), with the PI3K-AKT and FoxO pathways enriched with more core targets. The PI3K-AKT pathway regulates FoxO activity *via* AKT-mediated phosphorylation. Thus, the PI3K/AKT/FoxO signaling pathway is likely a key mechanism driving the synergistic effects of VPA and ALA combination therapy in ALS, which will be further investigated in our subsequent mechanistic validation.

### 3.5 Determination of core targets for the synergistic effect of VPA and ALA in ALS treatment

**FIGURE 6:**
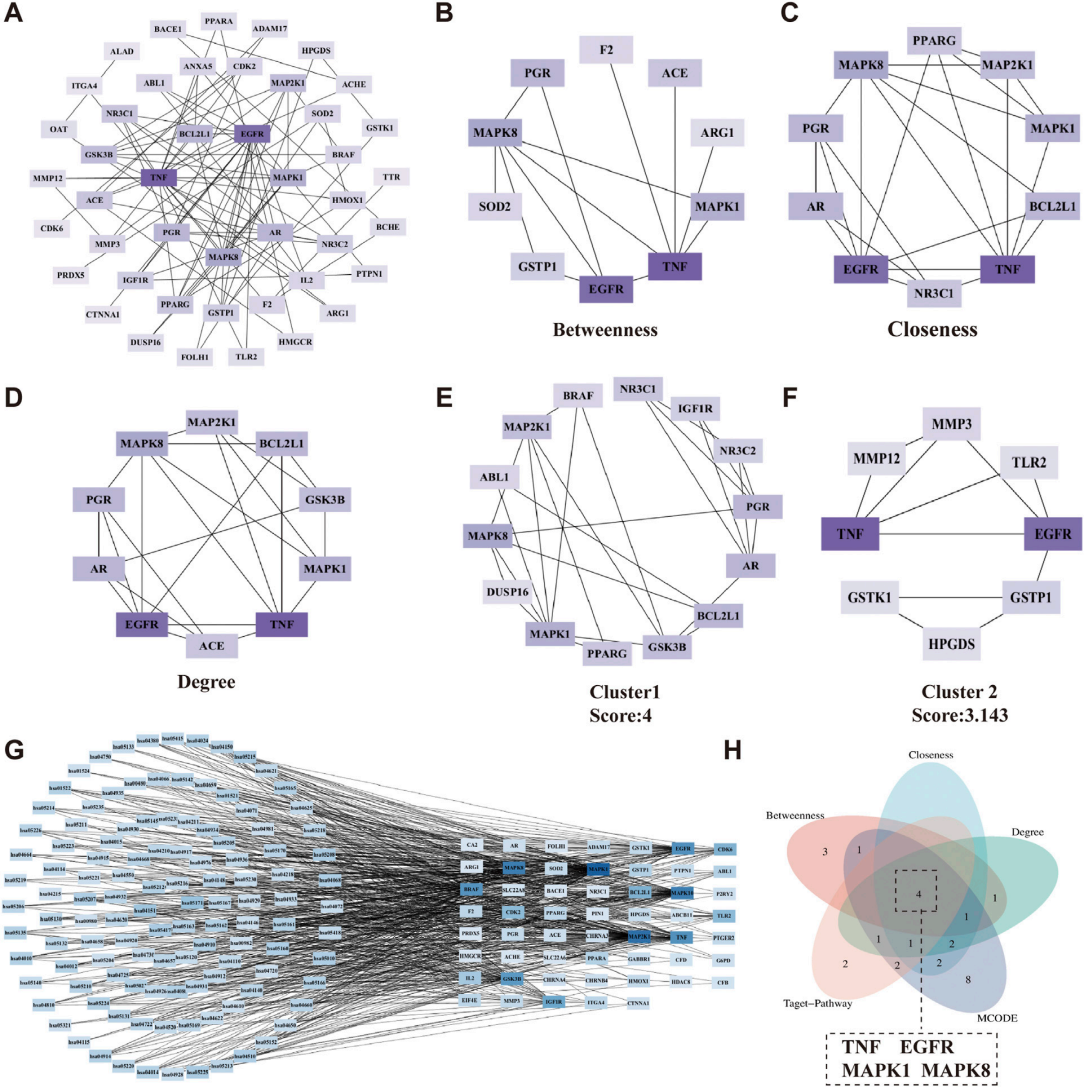
Determination of core targets for the synergistic effects of VPA and ALA in ALS treatment. Note: **(A)** The 45 synergistic effect targets were categorized based on their Degree values. Targets with Degree ≥7 were assigned to the first layer (innermost circle), 3 ≤ Degree <7 to the second layer, and 1 ≤ Degree <3 to the third layer (outermost circle). **(B–D)** Top 10 targets ranked by Betweenness, Closeness, and Degree in topological analysis using the CytoHubba plugin. **(E,F)** Gene clusters identified through clustering analysis using the MCODE plugin. **(G)** The relationship between synergistic effect targets and their associated KEGG pathways. **(H)** Identification of core targets. **(A–G)** Color intensity corresponds to Degree, with darker colors indicating higher Degree.

We used the PPI network constructed by Cytoscape software to form a network structure with 45 nodes and 88 edges. Based on Degree ranking, targets were classified into three layers (Figure 6A). The top 10 targets ranked by Betweenness were EGFR, F2, MAPK1, ARG1, GSTP1, MAPK8, SOD2, PGR, TNF, and ACE (Figure 6B), while those ranked by Closeness included EGFR, NR3C1, AR, MAPK1, PPARG, MAPK8, MAP2K1, PGR, BCL2L1, and TNF (Figure 6C). The Degree-based ranking identified TNF, EGFR, MAPK8, MAPK1, BCL2L1, PGR, AR, GSK3B, MAP2K1, and ACE as the top 10 targets (Figure 6D). Notably, a greater Degree indicates a more essential function in mediating the synergistic effects of VPA and ALA in ALS therapy.

Clustering analysis, performed using the MCODE plugin with optimized parameters, revealed two distinct gene clusters (Figures 6E,F). To improve our understanding of the link between targets and signaling pathways, we constructed a “target-pathway” network based on 117 KEGG pathways and their corresponding enriched targets. Degree-based ranking within this network highlighted MAPK1, MAP2K1, MAPK8, MAPK10, TNF, EGFR, BRAF, GSK3B, IGF1R, and CDK6 as key targets (Figure 6G).

The genes obtained from the above three methods were displayed using a Venn diagram, revealing four overlapping core targets: TNF, EGFR, MAPK1, and MAPK8 (Figure 6H). The protein names, gene symbols, and UniProt IDs of the hub targets are listed in Table 1. These genes are likely key mediators of the synergistic effects of VPA and ALA in ALS treatment and warrant further mechanistic investigation.

**TABLE 1:** Hub targets of the synergistic targets of VPA and ALA combination therapy for ALS.

No.	Gene name	Uniprot ID	Protein name
1	**TNF** (TNFA, TNFSF2)	P01375	Tumor necrosis factor, TNF-alpha
2	**EGFR** (ERBB, ERBB1, HER1)	P00533	Epidermal growth factor receptor
3	**MAPK1** (ERK2, PRKM1, PRKM2)	P28482	Mitogen-activated protein kinase 1
4	**MAPK8 (**JNK1, PRKM8, SAPK1, SAPK1C)	P45983	Mitogen-activated protein kinase 8

### 3.6 Molecular docking analysis of VPA and ALA with core targets

The core synergistic targets of VPA and ALA for ALS treatment were identified through network pharmacology and bioinformatics as TNF, EGFR, MAPK1, and MAPK8. To predict their binding modes and evaluate binding affinities, molecular docking was performed between VPA, ALA, and these four target proteins. The magnitude of binding energy reflects the probability of interaction between the receptor and ligand. Typically, a binding energy below 0 kcal/mol suggests that the receptor and ligand can bind spontaneously without the need for external energy, with lower values reflecting a stronger binding affinity. More specifically, a binding energy of less than −4.25 kcal/mol suggests moderate binding activity (Chia et al., 2021), whereas a value below −5 kcal/mol indicates strong binding affinity between the receptor and ligand (Jiao et al., 2023; Yong et al., 2021).

**FIGURE 7:**
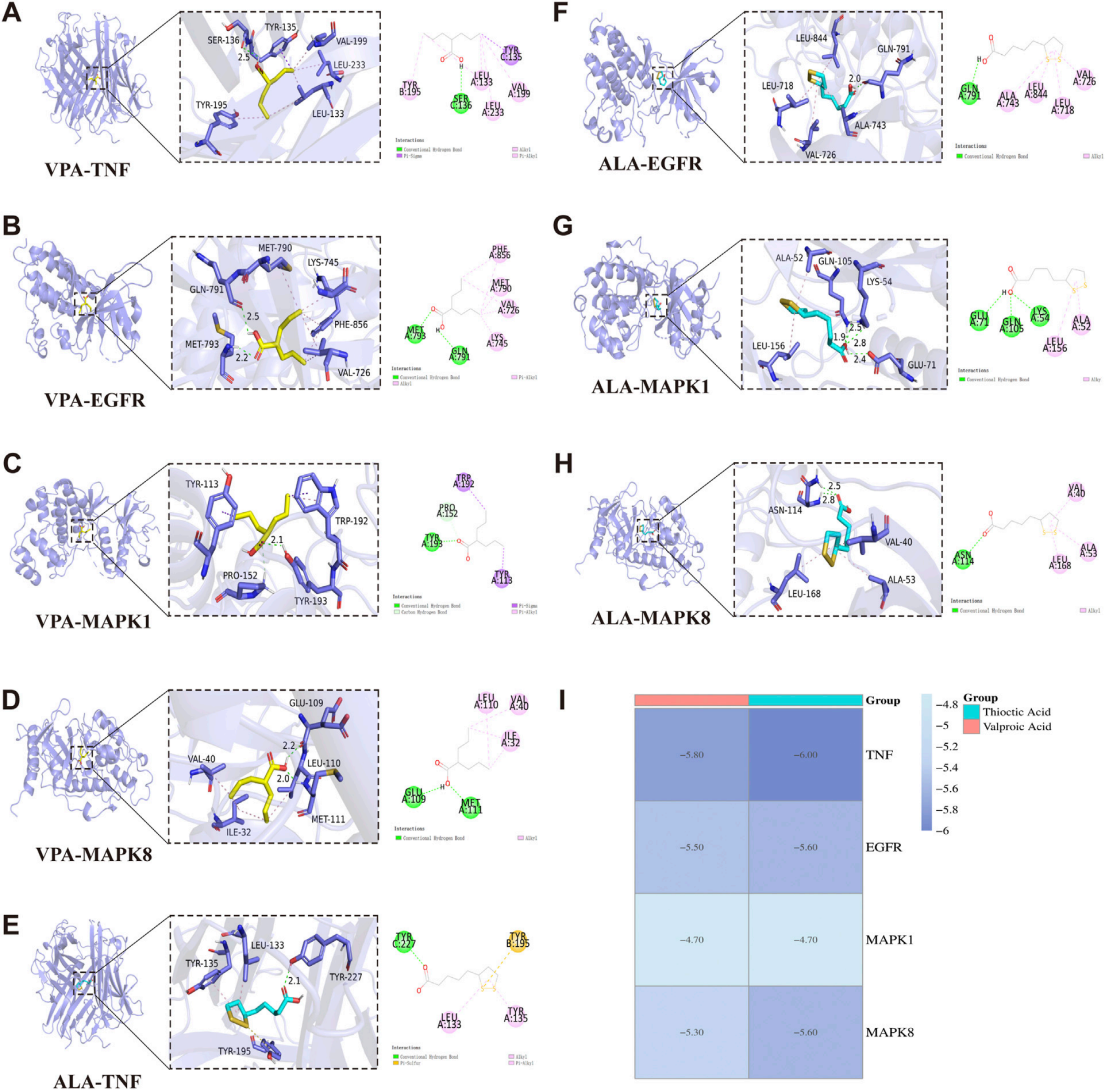
Molecular docking interactions between VPA, ALA, and core target proteins. Note: **(A–D)** Molecular docking models of VPA bound to TNF, EGFR, MAPK1, and MAPK8. **(E–H)** Molecular docking models of ALA bound to TNF, EGFR, MAPK1, and MAPK8. **(I)** Heatmap of molecular docking binding energies of VPA and ALA with core targets.

Molecular docking simulations were conducted separately for VPA and ALA against TNF, EGFR, MAPK1, and MAPK8. Figures 7A–H illustrates the interaction patterns of each molecular complex with the targets, while Table 2 summarizes the results. The binding energies of VPA with TNF, EGFR, MAPK1, and MAPK8 were recorded as −5.8, −5.5, −4.7, and −5.3 kcal/mol, respectively. For ALA, the binding energies with the same targets were −6.0, −5.6, −4.7, and −5.6 kcal/mol (Figure 7I). Since all binding energies were below 0 kcal/mol, both VPA and ALA exhibited favorable binding affinities with the core targets. Among them, ALA showed the highest affinity for TNF (−6.0 kcal/mol). Notably, while the binding energies of VPA-MAPK1 and ALA-MAPK1 were both −4.7 kcal/mol, exceeding −5 kcal/mol, the interaction involved considerable binding forces and multiple interaction bonds, indicating moderate binding strength that requires further validation.

**TABLE 2:** Binding energies (kcal/mol) of VPA and ALA with core target proteins.

Compound	Target	PDB ID	Column amino acid bindings	Affinity (kcal/mol)
VPA	TNF	7JRA	SER-136, TYR-195, LEU-133, LEU-233, VAL-199, TYR-135	−5.8
	EGFR	5UG9	MET-790, GLN-791, MET-793, VAL-726, PHE-856, LYS-745	−5.5
	MAPK1	6QAL	TYR-113, PRO-152, TYR-193, TRP-192	−4.7
	MAPK8	4L7F	VAL-40, ILE-32, MET-111, LEU-110, GLU-109	−5.3
ALA	TNF	7JRA	TYR-135, LEU-133, TYR-227, TYR-195	−6.0
	EGFR	5UG9	LEU-844, GLN-791, LEU-718, VAL-726, ALA-743	−5.6
	MAPK1	6QAL	ALA-52, LEU-156, GLU-71, LYS-54, GLN-105	−4.7
	MAPK8	4L7F	ASN-114, LEU-168, VAL-40, ALA-53	−5.6

Overall, molecular docking confirmed the strong binding abilities of VPA and ALA to the core targets, reinforcing the hypothesis that the synergistic effects of VPA and ALA in ALS treatment may be mediated through their interactions with TNF, EGFR, MAPK1, and MAPK8.

### 3.7 MR analysis

**FIGURE 8:**
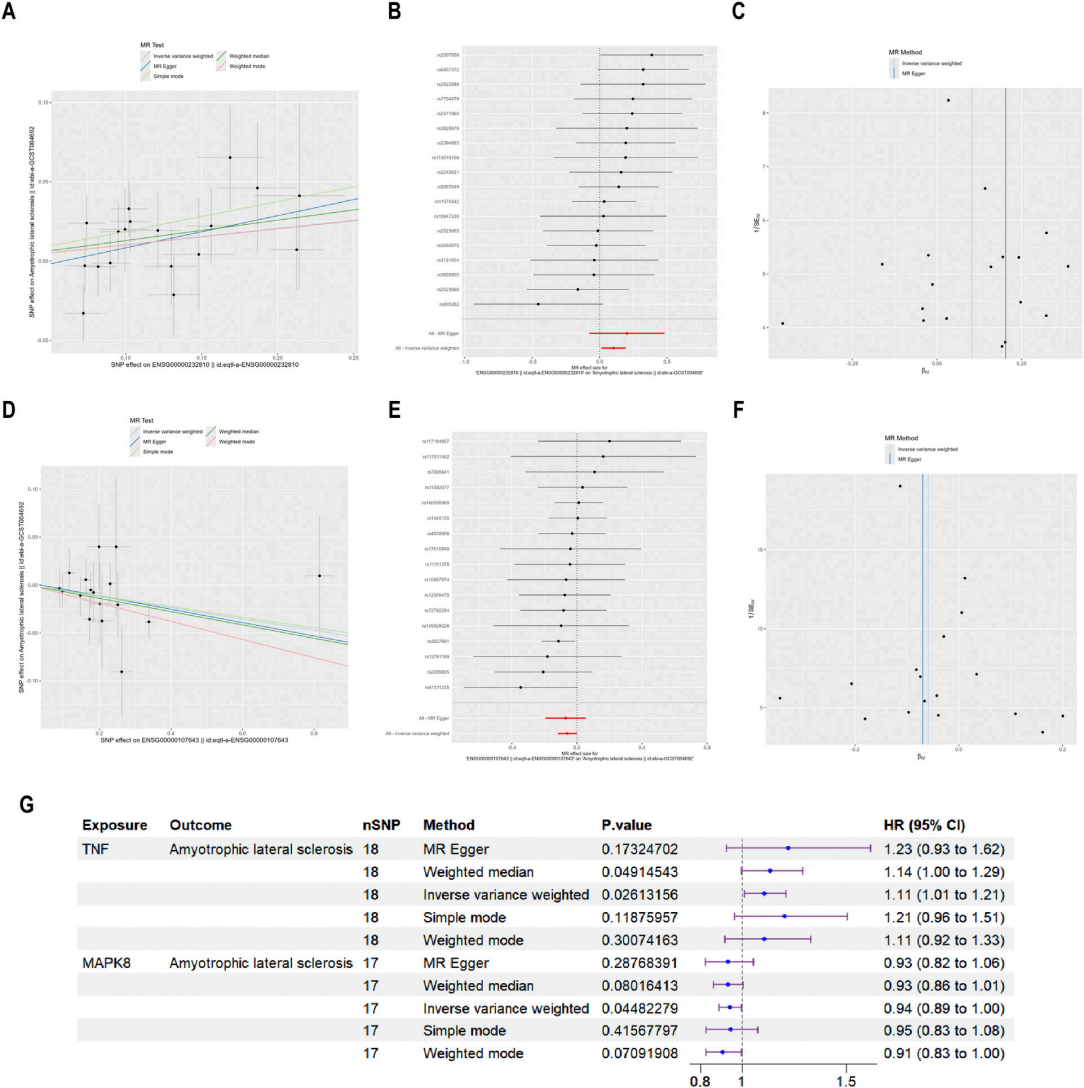
MR analysis of the causative connection among TNF, MAPK8, and ALS. Note: **(A,D)** Scatter plots depicting the causative influences of TNF and MAPK8 on the risk of ALS. **(B,E)** Forest plots illustrating the causal estimates of TNF and MAPK8 on the risk of ALS. **(C,F)** Funnel plots assessing the heterogeneity of MR estimates for TNF and MAPK8 in ALS risk. **(G)** Summary forest plot of MR analysis for TNF, MAPK8, and ALS.

The relationship between vulnerability to ALS and the four primary targets (TNF, EGFR, MAPK1, and MAPK8) was investigated using MR analysis. Among them, TNF and MAPK8 were significantly associated with ALS risk. The primary analytical approach employed was the IVW method (P < 0.05). MR analysis indicated a positive correlation between elevated TNF levels and an increased risk of ALS (OR = 1.108, 95% CI: 1.012–1.214, P = 0.026) (Figures 8A–C). Conversely, higher MAPK8 levels were found to be inversely related to ALS risk (OR = 0.943, 95% CI: 0.890–0.999, P = 0.045) (Figures 8D–F). A forest plot was created to visually represent the MR findings of TNF and MAPK8 concerning ALS (Figure 8G).

Pleiotropy and heterogeneity analyses for TNF and MAPK8 in ALS are summarized in Table 3. Both tests indicated no significant pleiotropy or heterogeneity (P > 0.05), confirming the stability and absence of bias in the IVs, and the MR-PRESSO test further confirmed this by not identifying any outlier SNPs. These findings reinforce the credibility and reliability of the causal inference between TNF, MAPK8, and ALS risk.

**TABLE 3:** Pleiotropy and heterogeneity analyses of TNF and MAPK8 in ALS causal inference.

Exp	Pleiotropy		Heterogeneity		MR-PRESSO p val
	MR Egger intercept	P val	MR Egger Q_p val	IVW Q_pval	
TNF	0.011965018	0.468069368	0.548437833	0.578981081	0.59
MAPK8	0.002506085	0.854236049	0.861064408	0.898778444	0.878

### 3.8 Combination treatment with VPA and ALA enhances cell viability in hSOD1^G93A^ NSC34 cells

**FIGURE 9:**
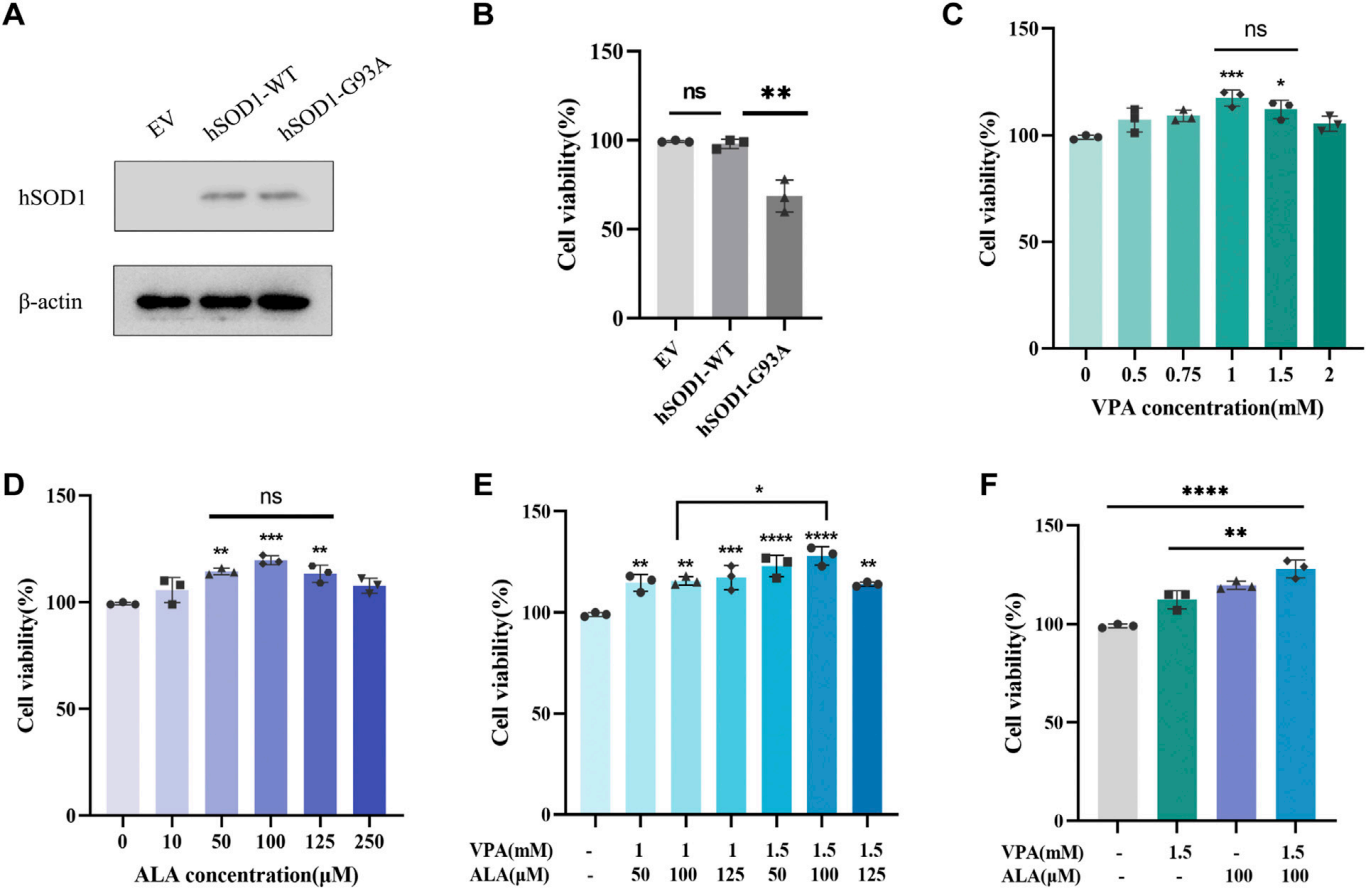
Effects of VPA and ALA, alone or in combination, on the viability of hSOD1^G93A^ NSC34 cells. Note: **(A)** WB analysis of hSOD1 protein levels in hSOD1^G93A^ NSC34 cells. **(B)** Viability of hSOD1^G93A^, hSOD1^WT^ and EV cells was measured by the CCK8 assay, **P < 0.01 compared with hSOD1^WT^ cells. **(C–E)** CCK-8 assay assessing cell viability in hSOD1^G93A^ NSC34 cells subjected to different doses of VPA **(C)**, ALA **(D)**, or their combination **(E)** for 24 h *P < 0.05, **P < 0.01, ***P < 0.001, ****P < 0.0001, *versus* 0 μM (mM). **(F)** Comparison of cellular viability after treatment with 1.5 mM VPA, 100 μM ALA, or their combination. Data are expressed as the mean ± SD (n = 3). Statistical analysis was conducted utilizing one-way ANOVA, succeeded by Tukey’s test.

WB analysis was conducted using an anti-hSOD1 antibody to confirm the successful transfection of hSOD1 in hSOD1^WT^ and hSOD1^G93A^ cell lines. The results demonstrated strong expression of human SOD1 protein in both hSOD1^WT^ and hSOD1^G93A^ cells, whereas no detectable expression was observed in the EV-NSC34 cells, indicating stable exogenous expression of human SOD1 protein in NSC34 cells (Figure 9A).

The vehicle-treated hSOD1^G93A^ cells showed significantly decreased cell viability when compared with the vehicle-treated EV or hSOD1^WT^ cells (Figure 9B). The impact of VPA on cell viability was assessed in hSOD1^G93A^ NSC34 cells subjected to different doses (0 mM–2.0 mM) for a duration of 24 h (Figure 9C). VPA concentrations of 1 mM (P < 0.001) and 1.5 mM (P < 0.05) significantly enhanced cell viability by 18.18% ± 3.50% and 13.13% ± 4.07%, respectively, compared to the control group. No substantial difference was noted between these two concentrations, leading to their selection for further combination therapy investigations.

The impact of ALA on cell viability was also evaluated in hSOD1^G93A^ NSC34 cells exposed to concentrations ranging from 0 μM to 250 μM for 24 h (Figure 9D). In comparison to the control group, ALA treatment at dosages of 50 μM (P < 0.01), 100 μM (P < 0.001), and 125 μM (P < 0.01) significantly increased cell viability to 115.1% ± 1.5%, 120.5% ± 2.1%, and 114.1% ± 4.1%, respectively. No substantial differences were observed among these concentrations, which led to their selection for further combination treatment investigations.

Using the optimal concentrations of VPA and ALA identified previously, we treated hSOD1^G93A^ NSC34 cells with various VPA + ALA combinations (1 mM + 50 μM, 1 mM + 100 μM, 1 mM + 125 μM, 1.5 mM + 50 μM, 1.5 mM + 100 μM, 1.5 mM + 125 μM) for 24 h. Subsequently, cell viability was evaluated to determine the impact of various treatments (Figure 9E). All tested combinations enhanced cell viability, with the 1.5 mM VPA +100 μM ALA combination exhibiting the greatest improvement in cell viability (P < 0.0001). While no notable change was seen between 1 mM and 1.5 mM VPA when used alone, the combination of 1.5 mM VPA and 100 μM ALA substantially surpassed the 1 mM VPA and 100 μM ALA combination (P = 0.0237).

To further determine whether the combination treatment was more effective than individual drug treatments, we compared the effects of 1.5 mM VPA +100 μM ALA with those of 1.5 mM VPA and 100 μM ALA alone on cell viability in hSOD1^G93A^ NSC34 cells after 24 h of treatment (Figure 9F). The combined therapy markedly enhanced cell viability relative to VPA alone (P = 0.0024), whereas the enhancement over ALA alone exhibited no significant difference. Significantly, in comparison to the control group, the combined treatment (1.5 mM VPA +100 μM ALA) significantly increased cell viability to 29.29% ± 4.60%, relative to ALA alone (20.87% ± 2.10%).

### 3.9 Combination treatment with VPA and ALA mitigates disease progression in ALS transgenic mice

**FIGURE 10:**
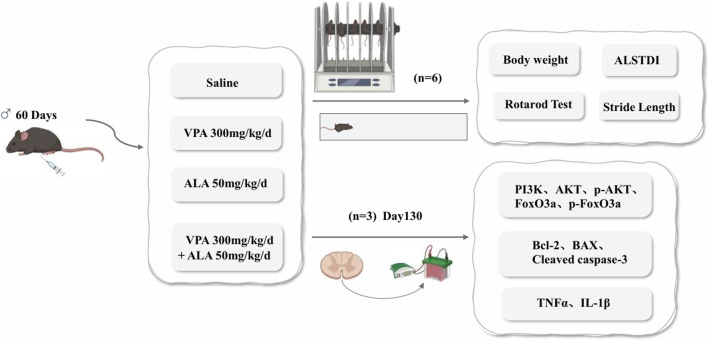
Schematic representation of the experimental design illustrating the neuroprotective effects of VPA and ALA in hSOD1^G93A^ mice.

**FIGURE 11:**
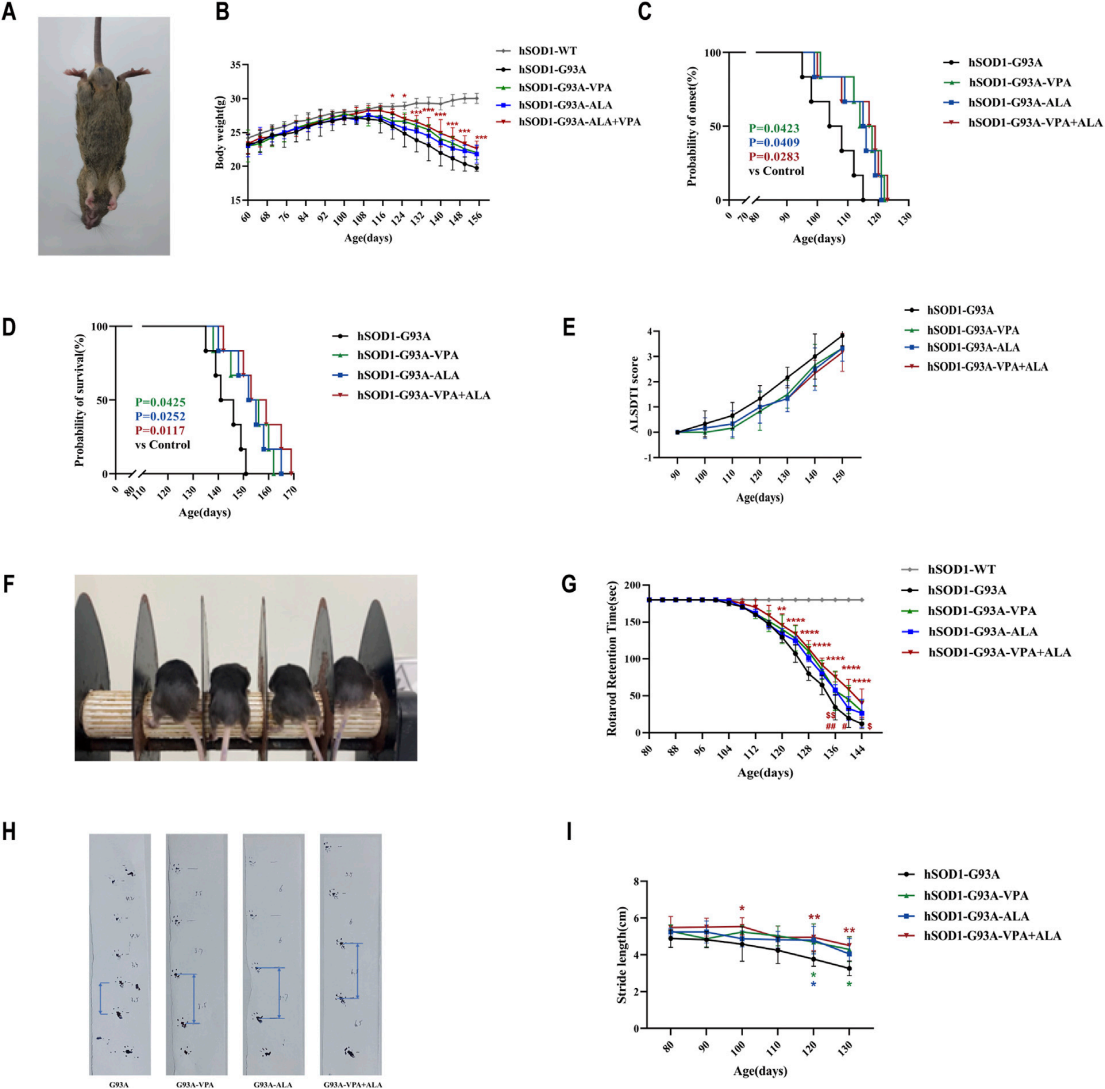
Effects of VPA, ALA, and their combination on hSOD1^G93A^ mice. Note: **(A)** Representative images of 130-day-old hSOD1^G93A^ mice. **(B)** Body weight. **(C)** Disease onset. **(D)** Survival curves. **(E)** ALSTDI scores. **(F)** Experimental setup for the rotarod test. **(G)** Rotarod performance. **(H)** Stride length measurement in 120-day-old hSOD1^G93A^ mice. **(I)** Stride length data. The Log-rank test was utilized to examine ALS onset and survival, whereas body weight, ALSTDI scores, rotarod performance, and stride length were evaluated using two-way ANOVA with subsequent Tukey’s test. Data are expressed as the mean ± SD (n = 6). *P < 0.05, **P < 0.01, ***P < 0.001, ****P < 0.0001 vs. control. #p < 0.05, ##p < 0.01 vs. VPA treatment group; $ p < 0.05, $$ p < 0.01 vs. ALA treatment group.

To investigate the potential medicinal applications of VPA and ALA, we examined their neuroprotective effects in ALS transgenic mice (Figure 10). At 30 days of age, Polymerase Chain Reaction was used to genotype hSOD1^G93A^ positive transgenic mice and their wild-type littermate controls. As the disease progressed, hSOD1^G93A^ mice developed hallmark ALS phenotypes, including rough and yellowed fur, progressive hind limb muscle atrophy, and a hunched posture. When suspended by the tail, their hind limbs failed to fully extend, as shown in Figure 11A.

Throughout disease progression, WT mice exhibited continuous body weight gain, while hSOD1^G93A^ mice demonstrated progressive weight loss. Although no significant statistical differences were observed between the single drug treatments (ALA or VPA) and the combination treatment, the combination of VPA and ALA exhibited a more pronounced protective trend in preventing body weight loss, suggesting a potential synergistic effect (Figure 11B). Both VPA and ALA treatments significantly delayed disease onset and extended survival in hSOD1^G93A^ mice. Monotherapy with VPA or ALA significantly postponed disease onset compared to the control group (VPA: 114.67 ± 7.74 vs. 105.33 ± 7.84, P < 0.05; ALA: 113.17 ± 8.06 vs. 105.33 ± 7.84, P < 0.05). Notably, combined VPA + ALA treatment resulted in a further delay in disease onset (114.50 ± 8.73 vs. 105.33 ± 7.84, P = 0.0283) (Figure 11C). Similarly, both VPA and ALA significantly prolonged survival compared to the control group (VPA: 152.17 ± 9.22 vs. 143.50 ± 6.19, P < 0.05; ALA: 153.00 ± 8.58 vs. 143.50 ± 6.19, P < 0.05), with the combined treatment showing an even greater extension of lifespan (156.33 ± 9.99 vs. 143.50 ± 6.19, P = 0.0117) (Figure 11D). Although no significant differences were found between the combination and monotherapy groups, the combination treatment showed a stronger trend toward improving disease progression and survival.

The ALSTDI scoring system revealed consistently lower scores in all treatment groups compared to the untreated hSOD1^G93A^ mice, indicating improved motor function and further supporting the neuroprotective effects of VPA and ALA (Figure 11E).

Motor coordination was further assessed using the rotarod test. While WT mice consistently maintained the maximum performance time of 180 s, hSOD1^G93A^ mice showed a progressive decline. Treatment with either VPA or ALA significantly increased fall latency compared to the control group, and combined treatment led to a further significant enhancement (P < 0.0001), with the combination showing a more pronounced effect than either monotherapy, suggesting a synergistic effect in improving motor performance (Figures 11F,G). Gait analysis using the stride length test further corroborated these findings. While untreated hSOD1^G93A^ mice exhibited a marked reduction in stride length over time, treatment with VPA, ALA, or their combination alleviated this decline, indicating preserved hind limb muscle function and coordination (Figures 11H,I).

In summary, these findings demonstrate that both VPA and ALA can prevent body weight loss, delay disease onset, extend survival, and improve neuromuscular function in hSOD1^G93A^ mice. Importantly, the combination of VPA and ALA showed superior efficacy compared to monotherapies, supporting its potential as a promising therapeutic strategy for ALS.

### 3.10 VPA and ALA synergistically enhance neuroprotection in hSOD1^G93A^ mice by activating the PI3K/AKT/FoxO3a pathway

Proteins were extracted from the lumbar spinal cord of 130-day-old hSOD1^G93A^ mice subjected to control, VPA, ALA, or the VPA + ALA combination treatments. The expression levels of PI3K, AKT, phosphorylated AKT (p-AKT), and its downstream targets, FoxO3a and phosphorylated FoxO3a (p-FoxO3a), were then evaluated *via* Western blot analysis.

**FIGURE 12:**
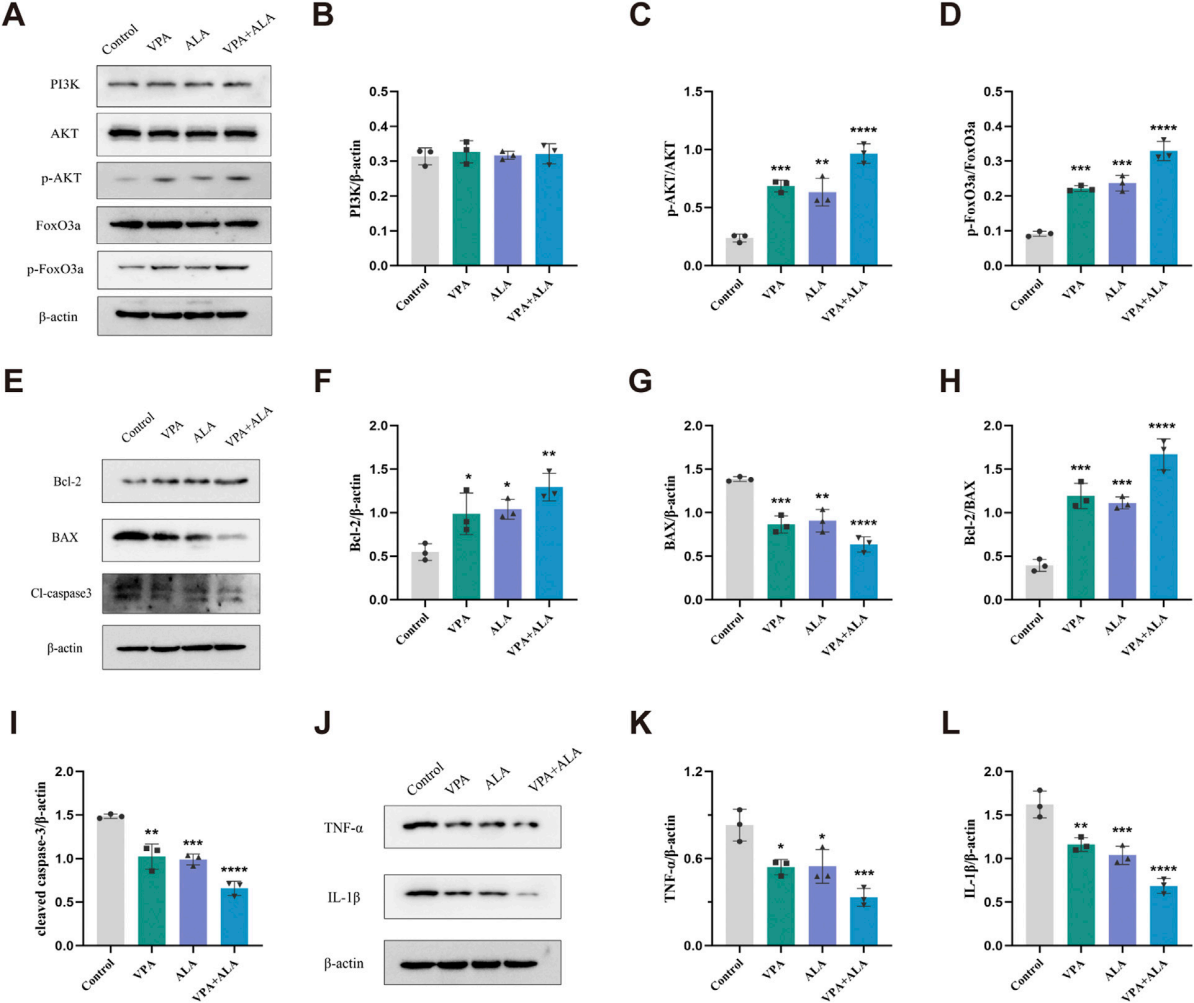
VPA and ALA stimulate the PI3K/AKT/FoxO3a signaling pathway in the spinal cord of hSOD1^G93A^ mice. Note: **(A)** WB analysis of PI3K, AKT, p-AKT, FoxO3a, and p-FoxO3a. Quantification is shown in **(B–D)**. **(E)** Bcl-2, Bax, and cleaved caspase-3 levels, with quantification in **(F–I)**. **(J)** TNF-α and IL-1β expression, with quantification in **(K,L)**. All expressions were normalized to β-actin. Data are presented as mean ± SD (n = 3) and analyzed by one-way ANOVA followed by Tukey’s *post hoc* test. *P < 0.05, **P < 0.01, ***P < 0.001, ****P < 0.0001 vs. control.

Although slight increases in PI3K and AKT expression were observed in the treatment groups compared to the control, these changes did not reach statistical significance. In contrast, the levels of p-AKT and p-FoxO3a were significantly elevated in the combination treatment group (P < 0.0001 for both) (Figures 12A–D), suggesting that VPA and ALA synergistically activate the PI3K/AKT/FoxO3a pathway, which may contribute to their observed neuroprotective effects in ALS.

Previous studies have reported that both VPA and ALA exert neuroprotective effects in various neurological disease models through activation of the PI3K/AKT pathway, thereby attenuating apoptosis and inflammation (Fahmy et al., 2024; Gurpur et al., 2009; Li and Sui, 2020). Building upon this evidence, we further examined the impact of VPA and ALA on key markers of apoptosis and inflammation in hSOD1^G93A^ mice.

Western blot analysis was conducted to evaluate the expression levels of critical apoptosis-related proteins, specifically Bcl-2, Bax, and cleaved caspase-3 (Figures 12E–I). Bcl-2 levels were significantly higher in the VPA, ALA, and combination treatment groups compared to the control group (P < 0.05, P < 0.05, P < 0.01, respectively) (Figure 12F). Bax expression was significantly reduced (P < 0.001, P < 0.01, P < 0.0001) (Figure 12G), along with cleaved caspase-3 levels (P < 0.01, P < 0.001, P < 0.0001) (Figure 12I). The Bcl-2/Bax ratio was significantly increased (P < 0.001, P < 0.001, P < 0.0001) (Figure 12H), supporting the anti-apoptotic effects of VPA and ALA.

We evaluated the anti-inflammatory effects of VPA and ALA by examining the expression levels of the pro-inflammatory factors TNF-α and IL-1β using WB (Figures 12J–L). In comparison to the control group, the VPA, ALA, and combination treatment groups demonstrated a significant decrease in TNF-α expression (P < 0.05, P < 0.05, P < 0.001, respectively). IL-1β expression was significantly reduced in these groups (P < 0.01, P < 0.001, P < 0.0001). The combination therapy led to more significant changes in apoptotic and inflammatory marker levels than the single-treatment groups, suggesting that the combined approach may provide a stronger therapeutic effect.

## 4 Discussion

This study employed network pharmacology to examine the synergistic and complementary effects of VPA and ALA in ALS, given the multi-target characteristics of both TCM and contemporary medication combinations. We aimed to elucidate the regulatory networks of VPA and ALA by integrating multi-omics data and molecular docking. This approach provides a theoretical foundation for repurposing these drugs in the treatment of neurodegenerative diseases.

Our study found that the combination of 1.5 mM VPA and 100 μM ALA significantly improved cell viability, outperforming either VPA or ALA monotherapy. This suggests a synergistic effect, where 1.5 mM VPA potentiates ALA’s protective role. Notably, although no significant difference in cell viability was observed between 1 mM and 1.5 mM VPA alone, the combination of 1.5 mM VPA and 100 μM ALA was more effective than 1 mM VPA combined with ALA. This implies that a higher concentration of VPA enhances ALA’s biological effects, potentially through modulation of cellular metabolism or activation of neuroprotective and antioxidant pathways. The observed therapeutic advantage of combination therapy likely arises from the distinct mechanisms of VPA and ALA, which not only enhance efficacy but also reduce potential side effects associated with single-drug treatments. Although this study identified several effective concentration combinations, further research is needed to refine the drug concentration range and examine the effects of different treatment time points to optimize the combined treatment regimen of VPA and ALA.

Through network pharmacology analysis, we identified 46 potential complementary targets for VPA, primarily involved in epigenetic regulation and histone deacetylation, which play a critical role in modulating gene expression and neuroprotection. As an HDAC inhibitor, VPA may influence chromatin structure and gene expression by regulating protein and histone deacetylation, thereby ameliorating ALS pathology (Bankole et al., 2022). These modifications contribute to the regulation of signaling pathways associated with neuroprotection, inflammation, and cell repair, providing a mechanistic basis for drug target exploration. For ALA, 75 potential complementary targets were identified, highlighting its role in mitigating oxidative stress and immune responses, which are critical for alleviating neuroinflammation and cellular damage. Furthermore, 68 shared targets were associated with the synergistic effects of VPA and ALA, with the PI3K/AKT and FoxO signaling pathways emerging as key regulators in ALS. These findings suggest that the combination therapy exerts multi-target effects, addressing both epigenetic dysregulation and oxidative stress-related mechanisms.

In our study, MR analysis indicates a potential causal relationship between genetically predicted elevated TNF levels and an increased risk of ALS, underscoring TNF as an attractive target for treatment. Our study revealed a potential regulatory role of VPA and ALA in EGFR-related signaling pathways in ALS. KEGG enrichment analysis showed that the combined treatment was significantly associated with both the ErbB signaling pathway and the EGFR tyrosine kinase inhibitor resistance pathway. These findings suggest that VPA and ALA may exert neuroprotective effects by modulating EGFR-mediated signaling. Nevertheless, the exact role of EGFR in ALS pathogenesis remains unclear and warrants further investigation, including the characterization of downstream signaling alterations and the direct effects on motor neuron survival.

MAPK1, or extracellular signal-regulated kinase 2 (ERK2), is integral to the pathogenesis of ALS, primarily due to abnormal phosphorylation or excessive activation. The study shown that VPA and ALA exert regulatory effects on MAPK1, potentially alleviating MN injury by modulating oxidative stress and neurotoxicity (Boeckeler et al., 2006; Hao et al., 2004; Marsh et al., 2005; Zhou et al., 2011). However, the precise role of MAPK1 in the progression of ALS remains to be further investigated, including changes in its downstream signaling pathways and its direct impact on MN survival, in order to clarify whether the ERK signaling pathway serves as a primary target through which VPA and ALA exert their neuroprotective effects.

MAPK8 also referred to as c-Jun N-terminal kinase 1 (JNK1), is responsible for regulating apoptosis, stress response and various physiological processes. In ALS, the activation of JNK is triggered by endoplasmic reticulum stress resulting from protein misfolding. JNK activation is closely linked to SOD1 aggregation and neuronal death, which further aggravates disease progression (Bhinge et al., 2017). In our study, Two-sample Mendelian Randomization analysis indicated that genetically predicted reduced MAPK8 expression was substantially correlated with an increased risk of developing ALS. Future studies should further investigate the dynamic regulation of MAPK8 during ALS progression and assess its long-term impact on neuronal survival to evaluate its viability as an option for therapy.

**FIGURE 13:**
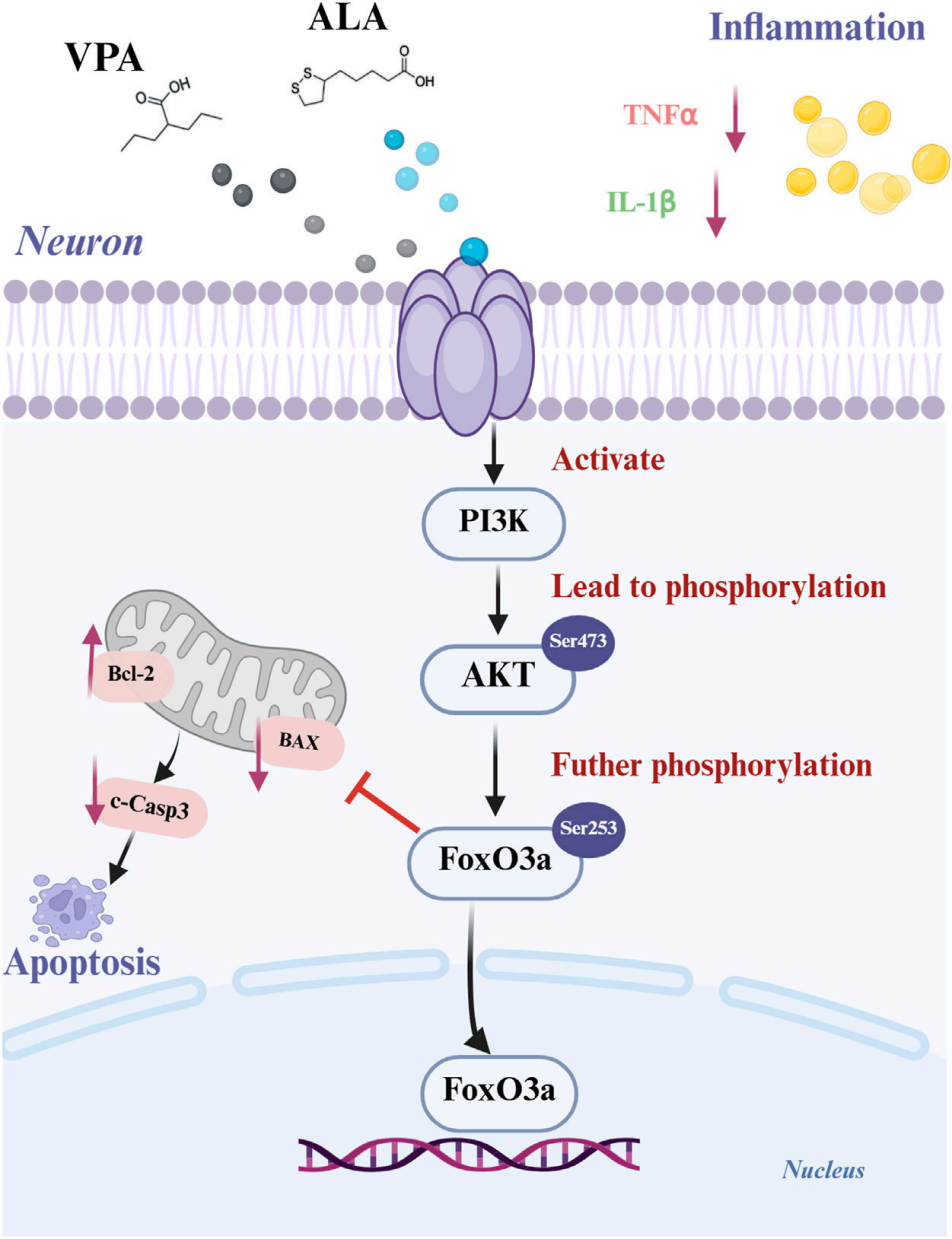
Schematic representation of the synergistic mechanism of VPA and ALA in ALS treatment. Created with BioRender.com.

The PI3K-AKT signaling pathway is essential for maintaining neuronal viability and providing neuroprotection. FoxO1 and FoxO3a of The Forkhead box O(FOXO) transcription factor family are the most deeply studied. Particularly, FoxO3a has been associated with the regulation of stress responses in MN (Oli et al., 2021). Recent evidence suggests that dysregulation of the AKT/FoxO3a signaling pathway may contribute to ALS pathogenesis (Halon-Golabek et al., 2018). Mechanistically, activation of AKT *via* phosphorylation at Thr308 and Ser473 leads to FoxO3a inhibition through phosphorylation at Thr32 and Ser253. While the exact function of FoxO signaling in ALS has not yet been thoroughly clarified, research indicates that its dysregulation may lead to disease progression. Depending on the cellular environment, FoxO activation can either enhance MN survival or worsen neuronal degeneration (Daneshafrooz et al., 2022). Therefore, further studies are required to elucidate the interactions among FoxO signaling components and to pinpoint potential therapeutic targets within this pathway. Previous studies have demonstrated that VPA and ALA activate AKT *via* the PI3K signaling pathway. A detailed analysis was conducted on the expression levels of PI3K, AKT, p-AKT, and their downstream targets, FoxO3a and p-FoxO3a (Ser253). The results demonstrate that VPA and ALA exert neuroprotective effects in ALS through the modulation of apoptosis and inflammatory responses. The PI3K/AKT and FoxO signaling pathways play a vital part in mediating these effects, implying their potential as therapeutic targets for ALS treatment in the future (Figure 13).

The effectiveness of VPA or ALA monotherapy for ALS treatment is still debated in animal studies (Andreassen et al., 2001; Rouaux et al., 2007; Sugai et al., 2004), which may be attributed to differences in ALS disease models, administration timing, dosage, and animal batches, among other factors. Our study demonstrated that VPA and ALA therapy modified motor performance and raised the lifespan of hSOD1^G93A^ transgenic mice, with combination therapy demonstrating superior efficacy compared to monotherapy. The neuroprotective effects of VPA and ALA presumably mediate several pathways, which may together contribute to their synergistic effects found in transgenic animals. However, while these results are promising in preclinical models, their clinical translation remains uncertain. Unlike experimental models, ALS patients often receive treatment at advanced disease stages, when MN degeneration is already extensive. Further studies are needed to determine whether combination therapy retains its efficacy in a clinical setting. Additionally, sex-specific differences have been reported in disease progression among hSOD1^G93A^ transgenic ALS mouse models (Sugai et al., 2004), and the influence of sex on ALA bioavailability and plasma concentration remains inconclusive (Hermann et al., 2014). Future studies should include larger sample sizes and consider potential sex-based differences in therapeutic responses.

Currently, therapeutic options for ALS remain limited, largely due to their high costs and limited accessibility. Given the multifactorial nature of ALS and the limitations of existing treatments, we explored a combination strategy using the repurposed drugs VPA and ALA, both of which have well-established safety profiles and are widely available. While this combination shows promising therapeutic potential, several limitations must be addressed, particularly the complexities of drug metabolism and potential interactions that may impact bioavailability. Moreover, the *in vivo* data should be interpreted with caution due to the limited sample size and statistical power. While the results consistently suggest neuroprotective effects of VPA and ALA, further validation in larger cohorts with more robust statistical analyses is essential to substantiate these findings. In addition, some relevant drug targets are not included in current network pharmacology databases, and the identification of ALS-related targets is an ongoing process. This may limit the comprehensiveness of our analysis. Although this study mainly focused on the PI3K/AKT/FoxO signaling pathway for experimental validation, other potentially relevant pathways were not explored, highlighting the need for more extensive mechanistic studies. Finally, considering the inherent heterogeneity of ALS, additional validation across a range of ALS models is crucial to evaluate the broader applicability of our results and facilitate their potential clinical translation.

Further research is needed to evaluate the pharmacokinetics and bioactivity of VPA and ALA, explore alternative delivery systems (e.g., nanoformulations), and investigate additional combinations, such as antioxidants or neurotrophic factors, to optimize therapeutic efficacy. Bridging preclinical findings to clinical trials in neurodegenerative diseases will be essential for validating these strategies and facilitating clinical translation.

## 5 Conclusion

In summary, this study demonstrates that the combination therapy of VPA and ALA exerts therapeutic effects in ALS by modulating multiple targets and pathways, with the synergistic effects primarily involving TNF, EGFR, MAPK1, and MAPK8. Genetic analyses show a significant association between elevated TNF expression and reduced MAPK8 levels, both of which correlate with an increased risk of ALS. The neuroprotective effects were confirmed in both the hSOD1^G93A^ cell and mouse models, with the combination therapy proving more effective than monotherapy. These effects are particularly mediated through modulation of the PI3K/AKT/FoxO3a pathway and anti-inflammatory and anti-apoptotic mechanisms.

## Data Availability

The raw data supporting the conclusions of this article will be made available by the authors, without undue reservation.
